# ‘Dysgu Cymraeg’ (Learn Welsh) – Supporting Psychiatrists to Increase Welsh Language Skills in the Workplace

**DOI:** 10.1192/bjo.2025.10282

**Published:** 2025-06-20

**Authors:** Dafydd Huw, Alka Ahuja, Oliver John, Laura Wyn Jones

**Affiliations:** 1 Royal College of Psychiatrists Wales, Cardiff, United Kingdom; 2 National Centre for Learning Welsh, Carmarthen, United Kingdom>AFF>

## Abstract

**Aims:** The Welsh Government’s strategic framework for promoting the Welsh language in health and social care places a responsibility on providers to proactively offer services in Welsh.

For many Welsh speakers, accessing Welsh language services significantly improves their overall experience and health and wellbeing outcomes. However, people often find it difficult to access these services and are reluctant to ask for them.

All healthcare workers have the potential to make a difference. Our aim was to develop a programme to support psychiatrists to increase their Welsh language skills in the workplace.

**Methods:** In June 2024, the Royal College of Psychiatrists Wales (RCPsych Wales) collaborated with the National Centre for Learning Welsh (NCLW) to realise our ambition. Our members were surveyed to gather expressions of interest in accessing tuition. 43 responses were received (around 10% of our membership) covering all health boards. A broad range of specialities and grades were represented.

7 respondents described their Welsh language skills as ‘Foundational’, ‘Intermediate’ or ‘Advanced’. These were referred to the National Centre’s Increasing Confidence programmes offered in Welsh Health Boards The remaining 36 said they had no skills or were ‘Beginners’. RCPsych Wales and NCLW agreed to develop bespoke provision for this group.

We also hosted a members’ webinar on the importance of learning Welsh and our partnership with NCLW.

**Results:** Following a tender exercise conducted by NCLW in December 2024, the Centre’s provider in the North West was contracted to provide a 10-week Welsh language taster course for psychiatrists.

A welcome meeting was held on 29 January 2025, with the course starting a fortnight later. Members were offered the flexibility to choose from 1 of 5 classes per week. These were delivered virtually and free of charge, each lasting an hour. Participants’ progress was also tracked to inform further future tuition.

The collaboration between RCPsych Wales and NCLW builds on the Centre’s previous experience with other colleges, e.g. the Royal College of GPs, and offers a valuable case study for future engagement.

**Conclusion:** When people experience ill-health, it is vital that they are able to access care in the language that best meets their needs. As a result of ‘Dysgu Cymraeg’, more psychiatrists know how to say a few words in Welsh and have been inspired to continue their language journey. The programme supports professional development, positively impacts patients’ lives, and contributes to the Welsh Government’s vision of a million Welsh speakers by 2050.

